# Histology-Grounded Automated Plaque Subtype Segmentation in Intravascular Optical Coherence Tomography

**DOI:** 10.1016/j.jscai.2024.102524

**Published:** 2025-03-18

**Authors:** Paul Young, Drew Nolen, Thomas E. Milner, Alexandra Gruslova, Deborah Vela, Louis Maximilian Buja, Luis A. Diaz Sanmartin, Paul Rad, Marc D. Feldman

**Affiliations:** a Department of Computer Science, University of Texas at San Antonio, San Antonio, Texas; b Department of Biomedical Engineering, University of Texas at San Antonio, San Antonio, Texas; c Department of Surgery, Baylor School of Medicine, Houston, Texas; d Department of Pathology, The Texas Heart Institute, Houston, Texas

**Keywords:** artificial intelligence, histology, intravascular optical coherence tomography, machine learning, neural networks, plaque classification

## Abstract

**Background:**

Intravascular optical coherence tomography (IVOCT) adoption has been limited by the complexity of image interpretation. The interpretation of histologic subtypes beyond lipid, calcium, and fibrous is challenging to human readers. To assist and standardize IVOCT image analysis, we demonstrate an artificial intelligence algorithm based on a histology data set that identifies lipid pools, fibrofatty, calcified lipid, and calcified fibrous in human coronary arteries for the first time.

**Methods:**

Sixty-seven human coronary arteries were imaged with IVOCT within 24 hours after death and then underwent histologic examination. IVOCT images were coregistered and segmented into histologic subtypes: lipid pools, fibrofatty tissue, calcified lipid, and calcified fibrous tissue. Experiments regarding lipidic plaque included fibrofatty tissue, lipid pools, and calcified lipids. Experiments regarding calcium plaque included calcified fibrous and calcified lipid plaques. Optical coherence tomography images were lumen justified and cropped to a depth of 200 pixels (1 mm) to account for limited optical coherence tomography penetration depth. IVOCT segmentations from expert readers guided by histology were used to train segmentation neural networks.

**Results:**

For each data set, in addition to testing each of these subtypes individually, we trained and tested the model on the combined grouping of subtypes. Combined lipid subtypes achieved validation and test Dice (Sørensen-Dice coefficient) of 0.63 and 0.40, respectively, whereas combined calcium subtypes achieved validation and test Dice of 0.66 and 0.62, respectively.

**Conclusions:**

This histology-validated artificial intelligence algorithm driven by histologic subtypes can identify plaque subtypes not evident to a human reader. The reported algorithm can provide a fast solution to IVOCT image interpretation.

## Introduction

Arterial plaque analysis can aid diagnosis and treatment selection of cardiovascular disease. Studies have linked arterial plaque composition with cardiovascular vulnerability, particularly with lipids and their subtypes.^1^^,^^2^ Intravascular ultrasound and near-infrared spectroscopy are capable but limited in their ability to analyze tissue composition. The spatial resolution of intravascular ultrasound is inferior to intravascular optical coherence tomography (IVOCT) and infrared spectroscopy is directed primarily at lipid detection and lacks ranging capability.^3^^,^^4^ IVOCT has shown value in imaging and classifying histologic plaque subtypes due to its fine resolution of 10 μm.^5^, ^6^, ^7^

Some expert readers can accurately classify plaque,^8^, ^9^, ^10^, ^11^ but this interpretation of IVOCT images is often slow and error-prone. This plaque misclassification, particularly in lipid subcategories, limits clinical acceptance of IVOCT despite its finer resolution.^12^, ^13^, ^14^ There are often hundreds of B-scans in a typical IVOCT pullback of an in vivo coronary artery. This complicates the analysis of vessel composition during interventions. Often, the only observable differences between plaque types are subtle variances of signal attenuation and texture. Deep fibrous can be confused with lipid and calcium plaques due to scattering attenuation, whereas lipid and calcium both have the potential to cause significant signal decay leading to confusion.^12^ The attenuation and scattering that lipid produces also can hide features of plaque behind it such as the distinct borders of calcium. Distinguishing lipid and calcium IVOCT image subtypes, such as lipid pools, calcified lipid, and calcified fibrous tissue, is beyond the capabilities of some human readers.

There are several approaches that have been developed to automate IVOCT plaque classification. These approaches suffer from their own drawbacks such as no histological verification, modified IVOCT devices, user region of interest selection requirements, or the lack of capability to differentiate lipidic and calcific plaque and their subtypes.^15^, ^16^, ^17^, ^18^, ^19^ We seek to develop a fully automated artificial intelligence (AI) based on histology validation that can segment IVOCT images to detect lipid and calcium plaques, as well as their subtypes.

## Materials and methods

### Imaging and data collection

The data collection method reflects the approach taken by Baruah,^20^ as there is some overlap in the data used in both approaches. Histology collection, coregistration, and some annotations were carried over from the previous work, whereas many of the histology-based annotations used in the current approach were revised with closer pathologist collaboration. Arteries were collected from 42 organ donor human hearts within 24 hours of death and imaged with an Abbott ILUMIEN IVOCT system. These arteries were extracted and mounted on a measuring device to aid histology coregistration. A Luer lock “O” ring was fitted to seal the proximal end of the artery. The artery was then perfused with saline at 100 mm of Hg and inserted with an IVOCT imaging catheter. Silk sutures closed all large side branches. IVOCT B-Scans were captured with the Abbott ILUMIEN default catheter pullback velocity of 20 mm/s. The artery was marked at proximal and distal ends to aid histology coregistration.

### Histology

After imaging, the coronary arteries were submerged in a 10% formalin solution. After formalin fixation, a Faxitron MX-20 (Faxitron Bioptics LLC) radiographed the arteries and Cal-Rite (Richard Allan Scientific) decalcified them overnight when necessary. Arteries were paraffin-embedded with a Tissue Tek Vacuum Infiltration Processor (Sakura Finetek USA) and sliced into 2- to 3-mm thick rings. Each artery provided an average of 25 rings. Serial 5-μm thick histologic sections were cut for each ring at 150-μm intervals and stained with hematoxylin and eosin, and with modified Movat’s pentachrome.^21^

### Coregistration

The IVOCT and histology images were coregistered, and a team of expert readers with guidance from histology and 2 cardiovascular pathologists manually segmented the IVOCT images into components. Key IVOCT and histology images were coregistered based on fiducial landmarks (eg, side branches, lumen geometric features). The remaining images were matched based on the measured micron distance from these landmarks in both IVOCT and histology images. Anatomic landmarks such as arterial branches or calcification patterns further aided coregistration.

### Plaque classification

Plaques were divided more precisely than Yabushita et al’s^11^ traditional lipid, calcium, and fibrous categories. Histological definitions for plaque subcomponents are as follows: (1) lipid pool, coalesced extracellular lipid including necrotic cores; (2) calcified lipid, a lipid pool or necrotic core that has become calcified; (3) fibrofatty, a heterogenous mixture of fibrous tissue and lipid; and (4) calcified fibrous tissue, regions of the collagenous extracellular matrix that are calcified.

### Ground truth

A team of expert IVOCT readers segmented ex vivo IVOCT frames into lipid pool, calcified lipid, fibrofatty, calcified fibrous, and fibrous subtypes, based on histologic guidance as provided by 2 expert histopathologists (D.V., M.B.). In contrast to optical coherence tomography (OCT) readers, pathologists have demonstrated near-perfect agreement when reading the same histology.^14^ Segmentations of any unclear OCT frames were confirmed in group consensus between at least 1 OCT reader and 1 histopathologist. Hematoxylin and eosin was the primary stain used for plaque identification, with some supplement of Movat’s pentachrome stain in complex cases. The core data set for lipid training and validation selected to prevent excessive class imbalance in the data set was 296 frames from 24 hearts. These frames were split randomly into training, validation, and test splits at a rate of 70/15/15. For calcium experiments, the data set was expanded to 820 frames from 42 hearts to include additional frames with calcium to better detect calcium. The calcium data set was split 70/15/15 into training, validation, and test splits, respectively. Split percentages could not be exact as frames from the same artery were not split to avoid contaminating results.

### Segmentation and analysis

Optical coherence tomography images input into the neural network were lumen justified polar OCT images similar to the approach by Lee et al.^22^ These images were further cropped to 200 pixels (∼ 1 mm) to account for the limited penetration depth of OCT. Segmentation masks were split into 3 categories: exclusion, background, and target class. Exclusion demarked inside the lumen and beyond the attenuation boundary. Background included fibrous as well as other constituents not currently being trained and evaluated. Target was the class being trained and evaluated. Experiments with combined groups were performed with multiple labels combined into 1 before input to the model.

### Training

The model was trained for 60 epochs with a starting learning rate of 0.0001. Plateau learning rate reduction was performed with a factor of 0.5 and patience of 4. Tversky focal loss was used with parameters optimized by plaque type. Validation metrics were used to select final model weights from epochs and runs. Test results were run on the selected model. The Central Illustration illustrates the process of dataset collection, model training, and sample inference.

**Central Illustration:**
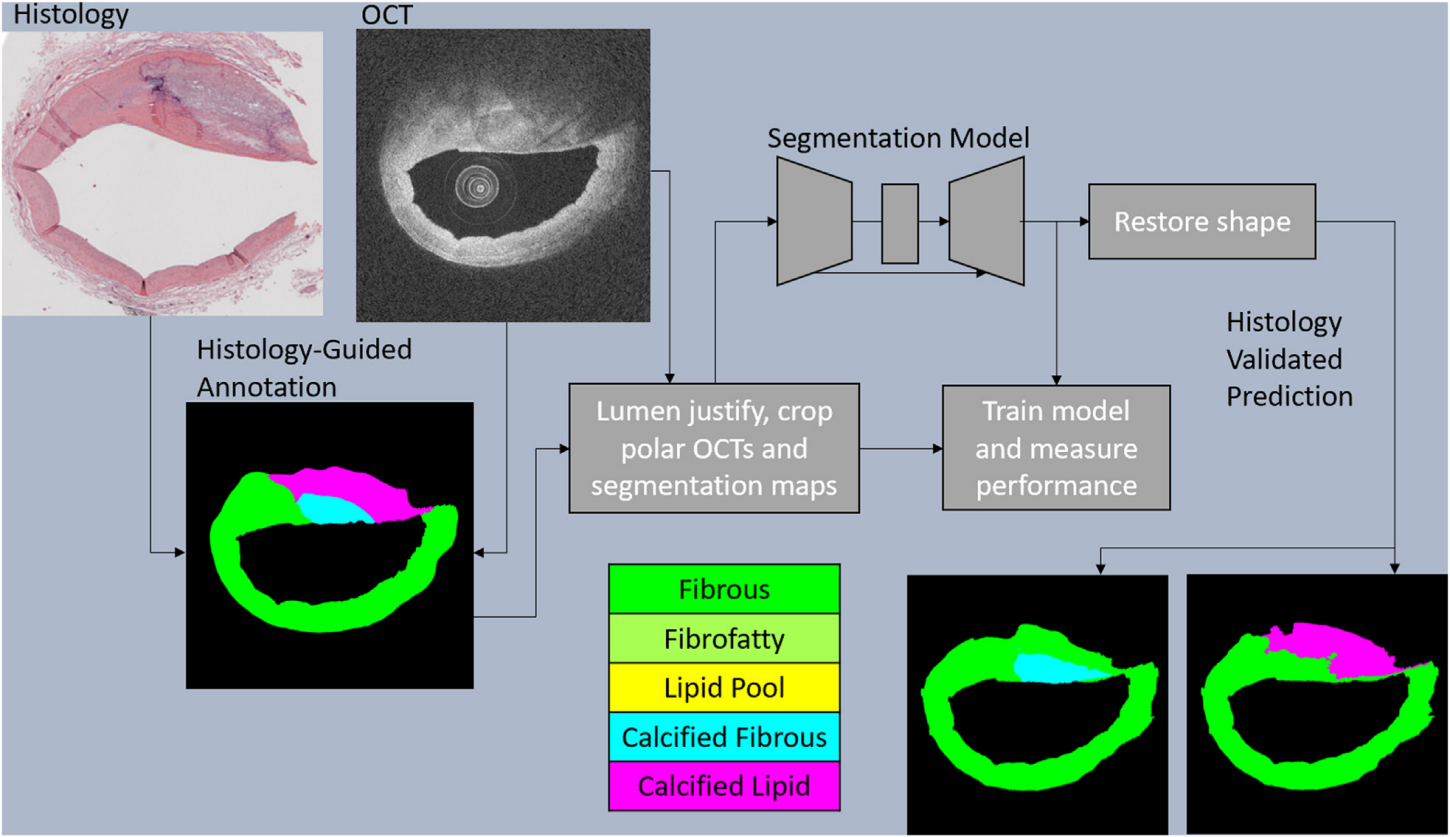
**Summaryworkflow.** Arteries used to develop this approach were imaged with Abbott ILUMIEN and analyzed with histology. Polar optical coherence tomography (OCT) images are flattened at the lumen and cropped to 200 pixels (∼ 1 mm) to focus on relevant regions. Images are then processed by the segmentation models for each plaque type to create the predictions for each plaque type. Predictions are then reshaped into their original form and converted to cartesian for viewing. OCT images are log-transformed for better visualization.

### Statistical methods

Dice score, a common segmentation metric, represents the Sørensen-Dice coefficient. This coefficient, which is equivalent to the F1 score used in classification problems, can be written as twice the cardinality of the set intersection over the sum of the individual set cardinalities. It weighs accurate predictions against both false positives and negatives. Sensitivity and specificity are based on A-line classification.^22^

## Results

Histology-based IVOCT annotations served as a ground truth to evaluate our segmentation approach. For the core lipid data set, 21 arteries from 16 hearts including 207 B-scans were used to train the model. After training, 4 arteries from 4 hearts including 46 B-scans, and 8 arteries from 6 hearts including 43 B-scans were used for the validation and test sets to test the lipidic classifier. In total, the lipid data set comprised 158 frames from 14 left anterior descending (LAD) arteries, 136 frames from 16 right coronary arteries (RCA), and 2 frames from 1 left circumflex (LCx) artery. Table 1 summarizes the performance of our approach for detecting various lipid subtypes on this data set.

**Table 1:** Lipidic subtype segmentation results.

Subtype	Validation			Test		
	Dice	Sensitivity	Specificity	Dice	Sensitivity	Specificity
Lipid pool	0.57	0.92	0.68	0.39	0.75	0.55
Calcified lipid^a^	0.44	0.73	0.93	0.21	0.65	0.82
Fibrofatty	0.04	0.92	0.32	0.02	0.84	0.20
Combined lipids	0.63	0.93	0.71	0.40	0.78	0.50

The table shows a summary of the segmentation results for lipidic plaque subtypes on the core lipid detection data set. Sensitivity and specificity are based on A-line classification. The combined category represents the prediction of all lipid subtypes together.

a Calcified lipid present in both lipid and calcium analysis due to its mixed composition.

For the expanded calcium data set, 40 arteries from 30 hearts including 577 B-scans were used to train the model. After training, 11 arteries from 9 hearts including 109 B-scans, and 16 arteries from 15 hearts including 134 B-scans were used for the validation and test sets to test the calcium classifier. In total, the expanded calcium data set contained 447 frames from 31 LAD arteries, 359 frames from 31 RCA arteries, and 14 frames from 5 LCx arteries. Table 2 summarizes the performance of our approach for detecting various calcium subtypes on this data set.

**Table 2:** Calcium subtype segmentation results on expanded calcium data set.

Subtype	Validation			Test		
	Dice	Sensitivity	Specificity	Dice	Sensitivity	Specificity
Calcified lipid^a^	0.44	0.76	0.83	0.43	0.79	0.85
Calcified fibrous	0.34	0.78	0.84	0.38	0.73	0.88
Combined calcium	0.66	0.78	0.92	0.62	0.73	0.94

The table shows the segmentation results of the 2 calcium subtypes as well as combined segmentation on the expanded calcium data set. Sensitivity and specificity are based on A-line classification. The combined category represents the prediction of all calcium subtypes together.

a Calcified lipid present in both lipid and calcium analysis due to its mixed composition.

Results for plaque subtypes are reported for validation/test sets in terms of Dice scores (Sørensen-Dice coefficient) and sensitivity and specificity calculated based on A-line classification. The following are plaque subtype results on the core lipid data set: for the lipid pool, the validation and test sets had Dice, sensitivity, and specificity values of 0.57, 0.92, 0.68 and 0.39, 0.75, 0.55, respectively. For fibrofatty tissue, the validation and test sets had Dice, sensitivity, and specificity values of 0.04, 0.92, 0.32 and 0.02, 0.84, 0.20, respectively. For calcified lipids, the validation and test sets had Dice, sensitivity, and specificity values of 0.44, 0.73, 0.93 and 0.21, 0.65, 0.82, respectively. For the combined detection of these plaque types, the validation and test sets had Dice, sensitivity, and specificity of 0.63, 0.93, 0.71 and 0.40, 0.78, 0.50, respectively.

**Figure 1:**
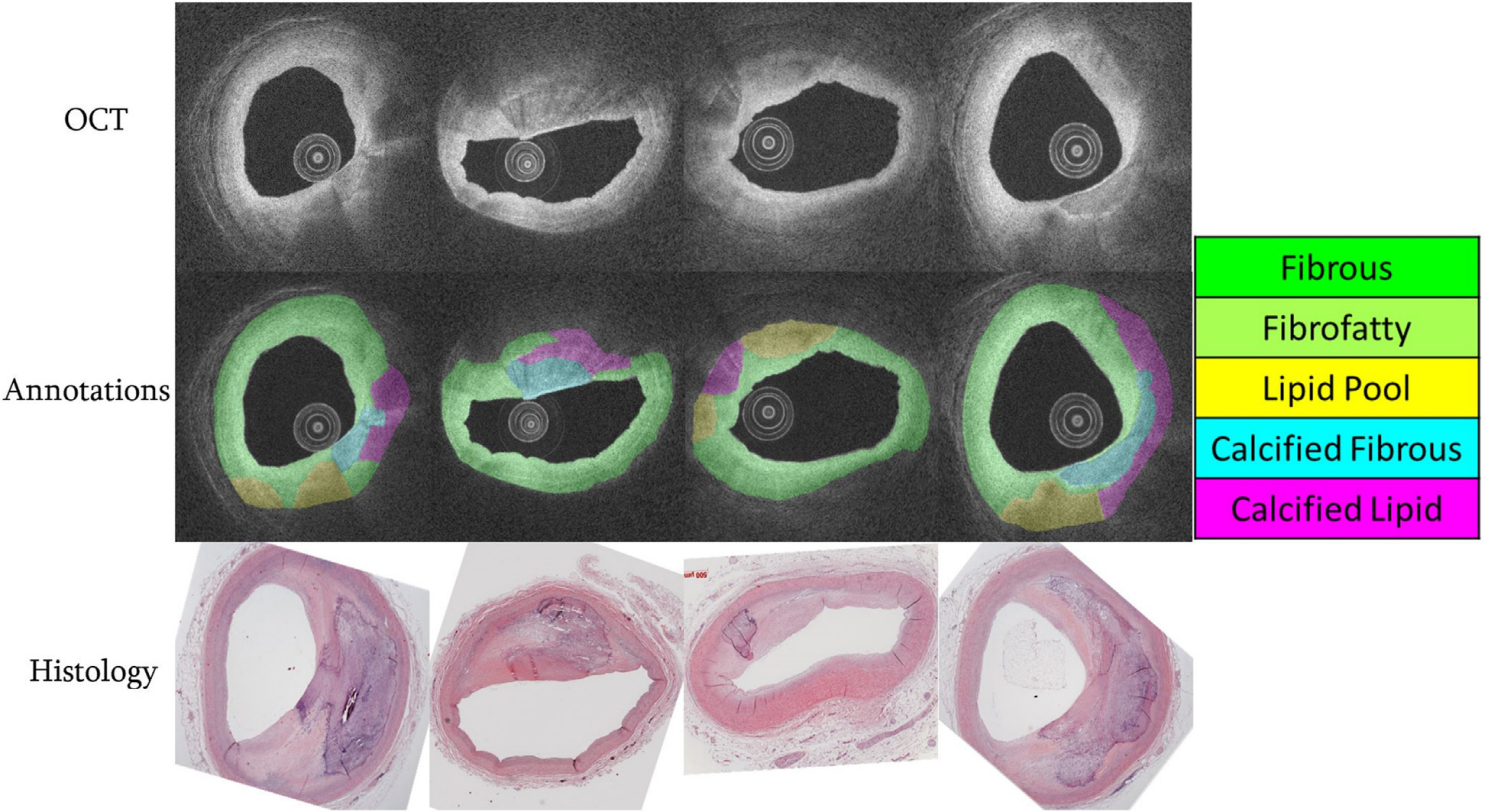
**Histological annotation of 4 plaque components.** To enable the detection of various plaque types and their subtypes, 820 frames from 67 arteries from 42 hearts were segmented into 5 plaque subtypes with the assistance of histology. Optical coherence tomography (OCT) images are log-transformed for better visualization.

The following are plaque subtype results on the expanded calcium data set: for calcified fibrous, the validation and test sets had Dice, sensitivity, and specificity of 0.34, 0.78, 0.84 and 0.38, 0.73, 0.88, respectively. For calcified lipids, the validation and test sets had Dice, sensitivity, and specificity of 0.44, 0.76, 0.83 and 0.43, 0.79, 0.85, respectively. For the combined detection of these plaque types, the validation and test sets had Dice, sensitivity, and specificity of 0.66, 0.78, 0.92 and 0.62, 0.73, 0.94, respectively. Figure 1 shows examples of 3 different categories of plaque detection in OCT images.

## Discussion

This neural network approach successfully segmented the plaque composition of IVOCT images into subcomponents beyond the traditional lipid, calcium, and fibrous^3^ for the first time. This included lipid pools, calcified lipids, and calcified fibrous tissue recognized by pathologists and OCT. These are subcomponents that can appear in OCT images of coronary atherosclerosis. However, they are not normally evident to IVOCT readers without reference to histology. This is an attractive application for AI in interventional cardiology, where human readers are unable to identify lipids and their subtypes, and calcium and its subtypes.

The complexity of reading and interpreting high-risk plaque in coronary arteries using IVOCT imaging is a common concern among interventionalists. The ability to characterize and classify atherosclerotic plaque in coronary arteries is largely verified by the extensive years of experience in the laboratory's use of the Abbott ILUMIEN system, which enhances the clinical applicability. Segmentation masks of the IVOCT images were done by using an established process where the IVOCT images were labeled according to each plaque type and subtype. After segmentation masks were developed and AI models trained, accuracy metrics, such as Dice scores, sensitivity, and specificity, were calculated. The results from these metrics show strong relevance toward clinical application. They can be easily integrated into the routine practice of interventionalists, which aids in more confident calcium and lipid, and their subtype identification and leads to more effective diagnoses and realistic prognoses. These more precise measurements and visualizations of plaque help physicians employ the optimal technique for the treatment of the target lesions. Treatments such as atherectomy, lithotripsy, or cutting balloons would modify or break up calcium plaques, improving successful stent expansion. In the future, the identification of lipid plaques and their risk for future rupture may lead to preemptive vulnerable plaque treatments.

Similar to Shibutani et al,^23^ as shown in Table 3,^23^ we report a plaque segmentation algorithm trained and validated with histological guidance. Histological grounding to evaluation metrics is critical because IVOCT complexity can lead to inaccurate readings—even among experts.^15^^,^^24^ These reading errors can affect both training and testing. Histology comparison reduces individual reader error as well as variance between readers resulting in more meaningful accuracy measurements.

**Table 3:** Combined plaque detection and histological comparison.

Plaque	This study						Shibutani et al^23^	
	Validation			Test			Validation	Test
	Dice	Sensitivity	Specificity	Dice	Sensitivity	Specificity	Dice	Dice
Calcium/fibrocalcific plaque	0.66	0.78	0.92	0.62	0.73	0.88	0.71	0.67
Lipid plaque/fibrous cap atheroma	0.63	0.93	0.71	0.40	0.78	0.50	0.48	0.62

The table summarizes the results of detecting combined calcium and lipidic plaques. We compare the closest matching categories with the other published histologically validated results of Shibutani et al.^23^ For this comparison, we matched our combined calcium plaque with their fibrocalcific plaque and our combined lipidic plaque category with their fibrous cap atheroma category of Shibutani et al.^23^ A-line sensitivity and specificity included for additional comparison. The similarity of our and Shibutani et al’s^23^ results is evident, both with lower Dice scores than studies using human expert readers as truth.

We experimented with various AI approaches to assess their impact on the ability to accurately detect plaque types. Because different plaque types have different intensity characteristics, we tested using image statistics such as attenuation maps as auxiliary inputs to inform the model. Ultimately, this approach did not demonstrate detection improvements. We compared U-Net and Transformer architectures, ultimately settling on a U-Net–type convolutional neural network. U-Net designs have been successful in situations with smaller data sets due to the shared weights from the fully convolutional structure reducing the tendency to overfit to the training data. Some additions to the basic design have been added such as attention parameters in the middle layers to capture longer distance relationships in the image and circular padding to connect the top and bottom edges in the polar OCT image inputs. These circular connections improved the continuity and accuracy of prediction across the polar image seam. Additionally, the higher-performing neural networks segmented 1 plaque type at a time rather than all together. Optimizing models to combine tissue types performs best because there is some overlap between features across multiple subtypes, such as distinct borders of calcified tissue.

A classification scheme based on subtypes of lipids and calcium enables a more nuanced IVOCT image segmentation. This facilitates addressing transitional states between the traditional plaque types and enables a more precise classification of mixed plaques. For instance, OCT may be able to recognize the progression of microcalcifications mixed into lipids, and its progression into calcium plate formation. One of the major benefits of using histology guidance for our annotations in this study was the ability to distinguish complex plaque types which today are only identifiable through pathological review. This is significantly more detailed than previous work which classified IVOCT into only 4tissue types (fibrous, lipid, calcified, and mixed plaque) based on IVOCT expert reader feature identification. Without histology, distinguishing these subtypes of lipid and calcified plaque types with IVOCT would be difficult.

Other studies have shown promising plaque detection metrics, but they lack a histological ground truth, making direct comparisons to the current work difficult. Without histology to compare against, the quality of the ground truth is difficult to assess without an intergroup comparison.^15^, ^16^, ^17^^,^^25^ Many of these studies which use expert readers as the ground truth for plaque classification have higher Dice scores than the 2 studies that utilized histology (the current study and Shibutani et al,^23^ Table 3). Although these expert annotations are referred to as “ground truth,” they are in fact an interpretation of the OCT image by readers who can make mistakes. Moreover, by their very nature of being difficult to detect, the plaques that readers miss will inevitably encompass the more difficult plaques. This simplification of the validation data is expected to lead to higher accuracy metrics due to the reduced difficulty of the task. This is demonstrated by the current study, the first to identify subcategories of lipids and calcium. When splitting larger plaque types into subtypes captured by histology such as calcified lipid and calcified fibrous tissue, the ground truth is more complex, resulting in lower Dice scores.

In the core lipid data set (Table 1) a distinct variance is noted between the accuracy measured on validation and test splits. This is due to the small size of the data set and the random sampling of the splits. This issue was addressed in the expanded calcium data set where arteries were split to evenly spread each subtype across the splits (Table 2). This is observed by comparing the calcified lipid results on the lipid data set to the calcified lipid results on the expanded calcium data set.

Our results also show a distinct difficulty in detecting fibrofatty tissue. Both fibrofatty and lipid pools produce a smooth OCT signal attenuation. Although lipid pools should attenuate more rapidly than fibrofatty due to the higher density of lipids which attenuates the signal quickly, their appearance varies somewhat as well. Because of the difficulty distinguishing these varying presentations of lipid pool from fibrofatty and the limited fibrofatty regions in our data set compared to lipid pools, our model has difficulty distinguishing between the 2. Future work can expand on the detection of more difficult plaques such as fibrofatty plaque and lipid-obscured calcium plaque.

Timely analysis is a key requirement to make effective use of this technology in a clinical setting. Compared to classifiers requiring manual region selection, approaches that can be fully automated, such as ours, present a speed advantage facilitating immediate use after IVOCT imaging. Using a NVIDIA V100 GPU, 250 images, representing a typical pullback, were predicted for 1 plaque type in 101 seconds. By implementing speed optimizations, this can be reduced further. Similar assessments using comparable models are capable of assessing an IVOCT pullback in <25 seconds.^26^

### Limitations

The vast majority of our images came from LAD and RCA coronary arteries. LCx arteries are more tortuous, making coregistration with histology more difficult. This may reduce accuracy when predicting plaque composition in LCx images. Additionally, the lack of external validation from outside data sets limits the assessment of the robustness of our approach. If OCT segmentation data sets become widely available, this will be more feasible to assess.

## Conclusion

We developed a novel automated algorithm that provides colorized segmented plaque in human coronary artery IVOCT images. We validated the algorithm’s ability to identify relevant subtypes of vulnerable plaque, including lipid and calcium plaque subtypes, utilizing histology. Clinically relevant specificity, sensitivity, and Dice metrics were achieved. Our study’s accuracy is supported by a 42-heart database, comprising hundreds of IVOCT images with coregistered histological slices. Compared to a validation process supported by expert readers, the ground truth provided by histology improved algorithm validation and overall design. Our study reports a fast, consistent, and clinically useful tool for IVOCT analysis and plaque characterization.
